# Decision-Making in Surgical Treatment for Posterior Inferior Cerebellar Artery (PICA) Aneurysm

**DOI:** 10.21315/mjms-05-2025-349

**Published:** 2025-12-31

**Authors:** Sarah Atiqah Mohd Zamri, Nurfaten Hamzah, Thinesh Kumaran Jayaraman

**Affiliations:** 1 Department of Neurosurgery, Tunku Abdul Rahman Neuroscience Institute, Hospital Kuala Lumpur, Kuala Lumpur, Malaysia; 2 Department of Neurosciences, School of Medical Sciences, Universiti Sains Malaysia, Health Campus, Kelantan, Malaysia

**Keywords:** posterior inferior cerebellar artery, aneurysm, bypass, endovascular, microsurgery

## Abstract

**Background:**

Posterior inferior cerebellar artery (PICA) aneurysm is rare. Endovascular treatment does not provide a decompressive effect, and the complex morphological features of PICA aneurysm make treatment approaches challenging. The objective of this study was to formulate a decision-making guide for PICA aneurysm according to location, based on an illustrative case and a review of the literature.

**Methods:**

From January 2022 until April 2023, a total of 157 aneurysm cases were treated using surgical and endovascular intervention. Fourteen patients were confirmed to have PICA aneurysm based on cerebral DSA/CTA. Demographic data, clinical characteristics, radiological findings, treatment plans, and outcomes were analysed. Outcomes were assessed using the modified Rankin Scale (mRS), with scores of zero to three considered good. Statistical analyses included Fisher’s exact test, Chi-square test, and McNemar test, where appropriate.

**Results:**

Of the total aneurysm cases, 8.9% were ruptured PICA aneurysms. Seven cases were located at the proximal PICA (P1 = 4 cases, P2 = 3), two cases at the mid PICA (P3), and five cases at the distal PICA (P4, P5). Out of 14 PICA aneurysms cases, nine were saccular, four were fusiform, and one was a dissecting aneurysm. Three patients underwent endovascular intervention (two saccular and one dissecting), while 11 underwent surgery (1 PICA–PICA *in situ* bypass with vertebral artery ligation, and 10 craniotomies with aneurysm clipping, with or without aneurysm reconstruction). Seven of 14 patients initially presented with good mRS scores, and at discharge, 10 patients had good scores. At discharge, two of three patients who had endovascular intervention had good mRS scores, and eight of 11 patients who went for surgery achieved good outcomes. Comparative analyses revealed no statistically significant associations between intervention type, aneurysm complexity, or location and outcome (*P* > 0.05). The McNemar test showed no significant difference between pre- and postoperative mRS scores (*P* = 0.25). However, a clinical trend toward improvement was observed, with the proportion of patients achieving good functional outcomes increasing from 50% before surgery to 71.4% at discharge.

**Conclusion:**

Favourable outcomes could be achieved when PICA aneurysm is treated with appropriate surgical strategies by experienced surgeons. Neurovascular surgeon must acquire knowledge of different surgical approaches and the technical skills required to perform these procedures.

## Introduction

Approximately 3% to 4% of all intracranial aneurysms are posterior inferior cerebellar artery (PICA) aneurysms (1). They can be saccular, fusiform, or dissecting in morphology, with the dissecting type generally carrying the worst prognosis (1, 2). The most common type of PICA aneurysm is fusiform. However, most of the ruptured PICA aneurysms are saccular (2). PICA aneurysm can arise from any of its five segments, with the vertebral artery (VA)—PICA junction being the most frequent site, while distal PICA aneurysm accounts for less than 30% (2). Compared with other intracranial aneurysms, PICA aneurysms more commonly display complex morphologies, making both surgical and endovascular interventions particularly challenging (1).

Although microsurgical treatments are complex and carry risks or morbidities, it should be noted that up to 79% of fusiform PICA aneurysms cannot be treated with coiling (1). Hence, surgical intervention, especially bypass surgery, remains an important technique in treating PICA aneurysms, as most display complex morphologies (1). Large retrospective studies have reported rupture rates for PICA aneurysms ranging from 77% to 88%. These aneurysms have a high tendency to rupture even at small size because the vessel wall is relatively fragile (3–6). They have a high recurrent rate within 48 hours, and the mortality rate is three times higher than that of anterior circulation aneurysms (7, 8). Due to their rarity and diversity in location and morphology, treatments for PICA aneurysms are considered very challenging.

Endovascular treatment has remained a popular treatment for PICA aneurysm, particularly in patients with poor clinical status, as it reduces brainstem as well as cranial nerve injury that can be caused by surgical intervention (4). However, endovascular treatment has limitations, including higher recurrence rate and lower occlusion rate, with reported infarction rates as high as 33% (9). In addition, it cannot offer a decompressive effect as surgical treatment does. Furthermore, the complex morphology of PICA aneurysms makes endovascular approaches difficult (9).

There are multiple options for PICA aneurysms, ranging from just simple clipping procedures to as complex interpositional bypass surgery. Acquiring the skills and knowledge required for these surgical techniques is particularly important for young and inexperienced surgeon.

### Objective

PICA aneurysms are rare and commonly non-saccular; however, the saccular type is the most common to rupture. Due to their heterogeneous and unique locations, surgical treatment plans must be carefully strategised. This study aimed to respectively evaluate all PICA aneurysm cases managed at our centre and to review relevant literature. The findings were interpreted in the context of our available facilities, equipment, and surgical experience to guide decision-making and refine surgical management strategies for PICA aneurysms. This work also aims to encourage neurosurgeons to acquire new skills and strengthen their expertise.

## Methods

**Figure 1:**
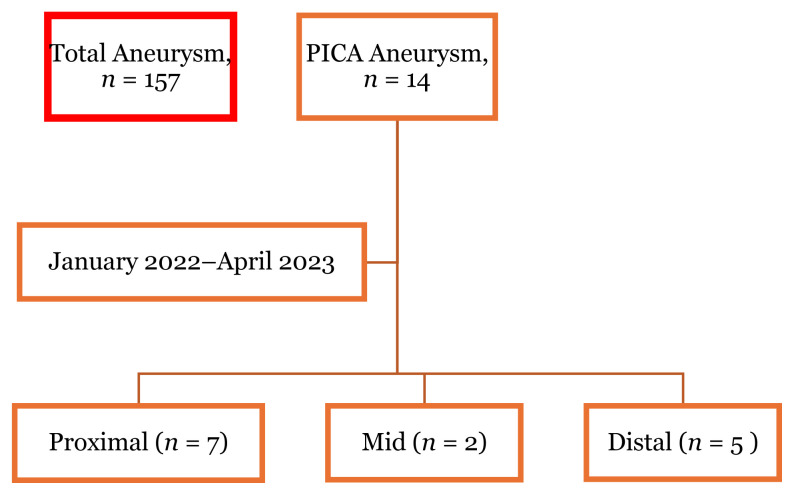
Total cases captured during the study period

From January 2022 until April 2023, a total of 157 cases of aneurysm were treated at our centre using either surgical or endovascular interventions. Among these, 14 patients were confirmed to have PICA aneurysm based on cerebral digital subtraction angiography (DSA) or computed tomography angiography (CTA). Demographic data, clinical characteristics, radiological findings, treatment plans, and outcomes were analysed. Figure 1 shows the total number of aneurysm cases captured during the study period.

The PICA branches from the fourth segment (V4) of the VA and is divided into five segments: i) anterior medullary (P1); ii) lateral medullary (P2); iii) tonsillomedullary (P3); iv) telovelotonsillar (P4); and v) cortical branches (P5). When aneurysm is present, these segments are further categorised as: i) proximal (VA–PICA junction, P1, P2); ii) mid (transitional, P3); and iii) distal (P4, P5) (2, 10). Complex PICA aneurysms are defined by the following characteristics: giant (≥ 25 mm) or large (10–25 mm) size, atherosclerotic wall structure, dissecting or fusiform morphology, wide neck, multiplicity, and recurrence after previous coiling or clipping (1). Neuroimaging used were CTA and DSA.

Craniotomies were performed via suboccipital or far-lateral, and if PICA–PICA *in situ* bypass was needed, the craniotomy was extended beyond the midline. Surgical decision-making and strategies were determined based on aneurysm morphology and location (see Figures 3, 6, and 10). Aneurysm clips used included Yasargil and Sugita models. When bypass was required, 3/8 round-body non-absorbable polyamide monofilament sutures were used.

Clinical outcomes were assessed using the modified Rankin Scale (mRS); scores of 0 to 3 were classified as good outcomes, while scores of 4 to 6 were classified as poor outcomes.

### Data Analysis

Data were analysed using IBM SPSS Statistics version 27. Descriptive statistics were used to summarise patient demographics. Continuous variables were assessed for normality using the Shapiro–Wilk test. Non-normally distributed continuous variables were expressed as median with interquartile range (IQR), while normally distributed variables were reported as mean with standard deviation. Categorical variables were presented as frequencies and percentages.

Comparative analyses were performed using Fisher’s exact test or Chi-square test for categorical variables. Comparisons were made between intervention type (surgical vs. endovascular) and outcome (good vs. poor mRS), aneurysm complexity (simple vs. complex) and outcome, as well as aneurysm location (proximal vs. distal) and outcome.

Comparison between pre-surgery and discharge outcomes (mRS categories) were assessed using the McNemar test for paired categorical outcomes. A *P*-value of < 0.05 was considered statistically significant. Given the small sample size, all analyses were considered as exploratory.

## Results

### Demographics and Clinical Characteristics

Among 157 aneurysm cases treated during the study period, 14 (8.9%) patients were identified as having PICA aneurysms. There were equal numbers of male and female patients (seven each). Patient ages ranged from 21 to 72 years old, with a mean of 50.64 ± 13.12 years. The aneurysm location distribution was as follows: seven cases at the proximal PICA (P1 = 4, P2 = 3), two cases at the mid PICA (P3), and five cases at the distal PICA (P4, P5). Laterality was evenly distributed, with seven cases on the left and seven on the right. Out of 14 PICA aneurysms, nine were saccular, four were fusiform, and one was dissecting. Three patients underwent endovascular intervention (two saccular and one dissecting), while 11 patients underwent surgical intervention. Among the surgical cases, one patient underwent PICA–PICA *in situ* bypass with VA ligation, and 10 patients underwent aneurysm clipping with or without reconstruction.

Of the total PICA aneurysms, seven were classified as simple and seven as complex. The median surgery duration was 4.8 hours (1), with a range of three to seven hours. The mean interval from presentation to definitive treatment was 3.29 ± 1.69 days, with a range of two to seven days. Before surgery, 7 of 14 patients (50%) had good neurological status (mRS 0–3). At discharge, 10 patients (71.4%) achieved good mRS scores. Among 14 patients, 8 of 11 (72.7%) surgically treated and 2 of 3 (66.7%) endovascularly treated patients had good outcomes at discharge. The summary of ruptured PICA aneurysm cases at our centre is presented in Table 1.

### Comparative Analysis

Comparative analyses were performed to explore potential relationships between intervention type, aneurysm complexity, and aneurysm location with clinical outcomes at discharge (Table 2). There was no statistically significant difference in outcomes between surgically and endovascularly treated patients (*P* = 1.00, Fisher’s exact test). Similarly, aneurysm complexity (simple vs. complex; *P* = 0.07, Fisher’s exact test) and aneurysm location (proximal vs. distal; *P* = 0.06, Chi-square test) were not significantly associated with functional outcomes. Although patients with proximal aneurysms demonstrated a trend toward better clinical outcomes, this trend did not reach statistical significance.

Changes in functional status before and after intervention were evaluated by comparing the mRS scores before surgery and at discharge using the McNemar test. A summary of patients’ mRS scores at both time points is presented in Table 3. The analysis showed no statistically significant change in the distribution of mRS categories before and after surgery (*P* = 0.25). However, a clinical trend toward improvement was observed, with the proportion of patients achieving good functional outcomes increasing from 50% before surgery to 71.4% at discharge. This finding likely reflects the limited sample size and the exploratory nature of the analysis.

## Discussion

This retrospective analysis highlights the clinical and surgical characteristics of PICA aneurysms managed at our centre.

Comparative analyses showed that aneurysm intervention, complexity, and location were not significantly related to patient outcomes (mRS). Patients with proximal aneurysms seemed to recover slightly better, possibly due to careful surgical planning and technique, but this difference was not statistically significant. These results are likely influenced by the small number of patients and variations between cases. The McNemar test revealed no statistically significant change in mRS scores before surgery and at discharge (*P* = 0.25), despite several patients demonstrating clinical improvement. The lack of statistical significance is likely due to the small sample size, which limits the test’s ability to detect differences. Despite this, the overall trend suggests that appropriate treatment, particularly timely surgery or endovascular intervention, can lead to meaningful recovery in many patients.

### Case Presentation

Hereby, the author would like to present some examples of cases that we have at our centre and the steps on making decision for treating this uncommon type of aneurysm.

#### Case 1: Proximal PICA Aneurysm (V4–PICA Junction Aneurysm)

**Figure 2:**
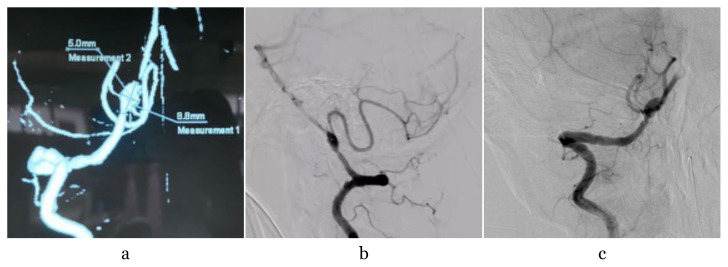
Cerebral digital subtraction angiography: a) 3D reconstruction; b) Right vertebral run, lateral view; c) Right vertebral run, anteroposterior view; a–c) Fusiform aneurysm at the right vertebral artery involving the origin of the right PICA, measuring 8.8 mm in length and 0.5 mm in diameter; The right PICA arises from the aneurysm sac

**Figure 3:**
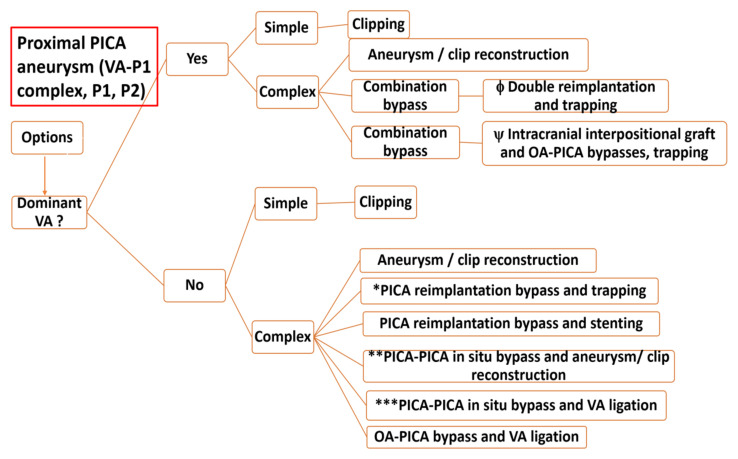
The algorithm for daecision-making for proximal PICA aneurysm (1, 11–14) *IR could not put a stent for this patient due to tight stenosis distal to the fusiform aneurysm **This type of surgery could not be performed for this patient because the aneurysm was bigger than expected from imaging and extended beyond the surgical field ***This type of surgery was performed for this patient ϕ Double reimplantation bypass = end-to-side anastomosis of the V3 VA with a radial artery graft, side-to-side anastomosis with P1 PICA, followed by end-to-end anastomosis with V4 VA ψ Intracranial interpositional bypass graft = end-to-side anastomosis of the V3 VA with a radial artery graft (RAG), connected by end-to-side anastomosis to the V4 VA (distal to aneurysm)

A 39-year-old Malay female presented to the emergency department with sudden severe headache and status epilepticus. Due to her low consciousness level upon presentation, she was intubated for airway and cerebral protection. CT brain imaging revealed diffuse subarachnoid haemorrhage (SAH) with hydrocephalus. Her neurosurgical grading was World Federation of Neurosurgical Societies (WFNS) Grade IV and Fischer Grade 3. Therefore, external ventricular drainage (EVD) was inserted. Cerebral CT angiography performed after surgery showed no abnormal vascular pathology. She was subsequently referred to our centre for cerebral DSA to evaluate any possible vascular pathology further, as the previous treating team did not have DSA facilities at their centre. Cerebral DSA showed a right VA–PICA junction fusiform aneurysm, involving the origin of the right PICA, with the PICA arising from the aneurysm sac (Figures 2a, 2b, and 2c). This aneurysm is located at a non-dominant vessel. The treatment options are shown in Figure 3.

**Figure 4:**
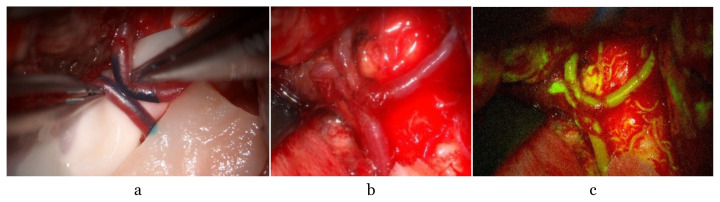
PICA–PICA *in situ* bypass: a) Both PICAs were approximated without tension, with the area of arteriotomy marked with methylene blue; b) PICA–PICA *in situ* bypass performed using non-absorbable polyamide monofilament 10/0 sutures; c) Flow assessment using indocyanine green (ICG) dye showed good flow (vascular doppler not shown)

**Figure 5:**
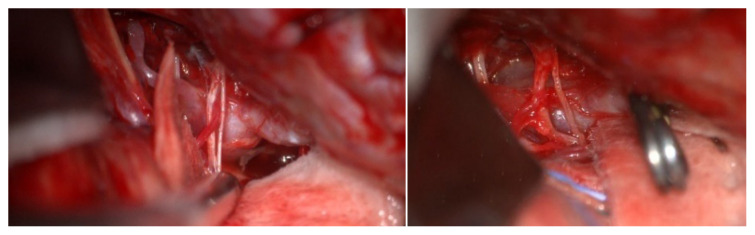
VA ligation proximal to aneurysm

After consultation with the interventional radiologist, stenting could not be performed in this case due to the presence of tight stenosis of the distal aneurysm. This led us to the next option, a PICA–PICA *in situ* bypass with aneurysm reconstruction. However, during the surgery, we noticed that the aneurysm was much bigger and longer than expected from imaging, extending beyond the surgical field. Consequently, reconstruction the aneurysm was not possible. This left two remaining options, and we proceeded with PICA–PICA *in situ* bypass (Figure 4) and VA ligation (Figure 5).

PICA–PICA bypass was preferred in this case because the distal PICA can tolerate prolonged temporary occlusion. Nevertheless, there is ongoing debate that OA–PICA bypass are better in terms of preserving the contralateral vessel from damage caused by temporary occlusion or insult.

#### Case 2: Mid PICA Aneurysm (P3 Aneurysm)

**Figure 6:**
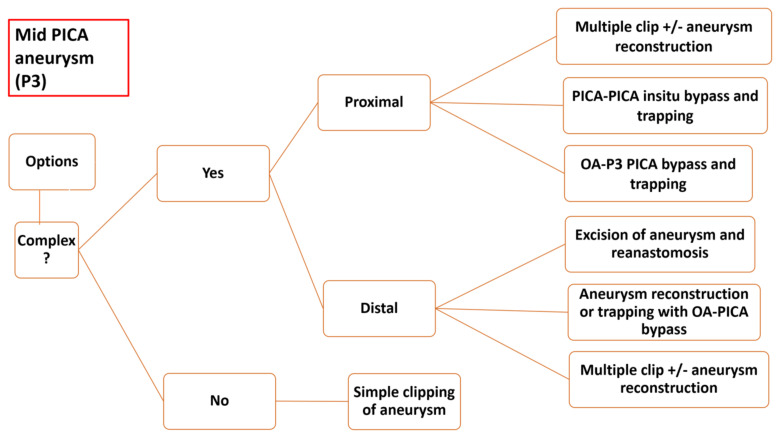
Algorithm on decision-making in mid PICA aneurysm (P3) (1, 11–14)

**Figure 7:**
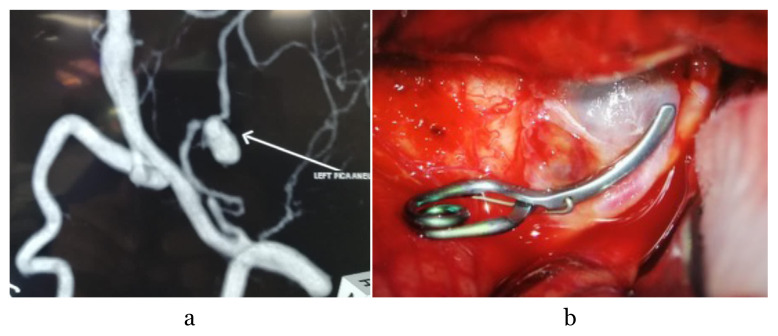
a) P3 PICA aneurysm; b) P3 PICA aneurysm with long curve clip on top of it

A 53-year-old Malay male presented to the emergency department with “the worst headache in his life.” He was hypertensive on arrival, and CT brain imaging showed thin SAH over the pre-pontine cistern and intraventricular haemorrhage (IVH) in the fourth ventricle. His grading was WFNS Grade I and Fischer Grade 2. CTA brain did not demonstrate any vascular pathology. Therefore, urgent cerebral DSA was performed, which revealed a fusiform aneurysm at the left proximal P3 segment of the PICA. Because the aneurysm had a wide neck and the surgeon was not familiar with bypass surgery at that time, the surgical plan was to put a long aneurysm clip or multiple tandem clips, sparing the parent vessel (Figure 6). Intraoperatively, a vascular doppler was used to confirm adequate flow (Figure 7).

#### Case 3: Distal PICA Aneurysm (P4, P5 Aneurysm)

**Figure 8:**
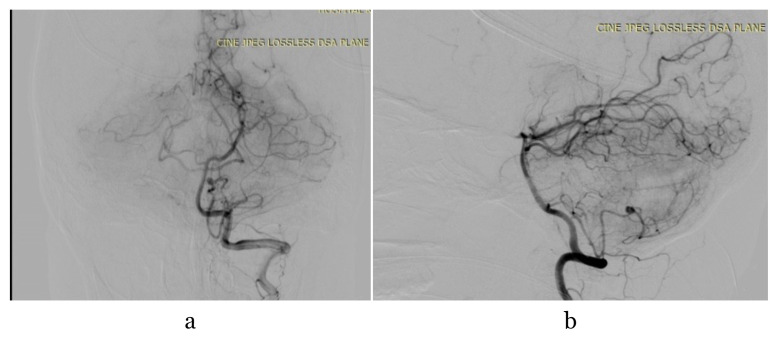
Cerebral DSA showing a saccular aneurysm at P4 PICA, projecting posterosuperiorly: a) Left vertebral run, anteroposterior view; b) Left vertebral run, lateral view

**Figure 9:**
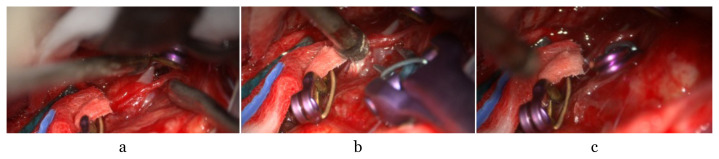
Titanium clip applied to P4 PICA aneurysm: a) Temporary clip applied to proximal and distal aneurysm; b) and c) Permanent clip applied across the neck of aneurysm

**Figure 10:**
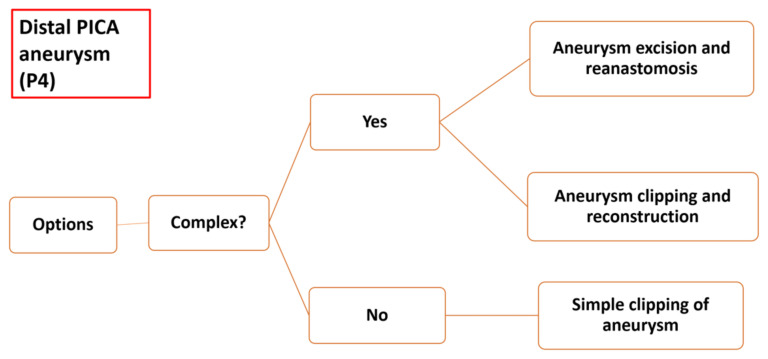
Algorithm on decision-making in distal PICA aneurysm (1, 11–14)

A 39-year-old Indian lady presented with severe headache and vomiting. Her grading was WFNS Grade I and Fischer Grade 4. Urgent CT brain imaging showed SAH with IVH and mild hydrocephalus. CTA demonstrated a distal left PICA saccular aneurysm at the telovelotonsillar segment (P4). Prior to cerebral DSA, the patient experienced persistent vomiting, and repeat CT brain imaging showed worsening hydrocephalus; therefore, EVD was inserted. Cerebral DSA revealed a distal left PICA saccular aneurysm measuring 3.3 × 2.5 × 2.2 mm, projecting posterosuperiorly (Figure 8). This was considered a simple aneurysm, and she was scheduled for far-lateral craniotomy with aneurysm clipping (Figures 9 and 10).

We presented an algorithm for decision-making in different parts of PICA aneurysms, with the aim of assisting in the selection of appropriate surgical treatment. Knowledge of the anatomical triangles in which the aneurysm is located also helps in understanding the surgical route and dissection of these aneurysms, in relation to the lower cranial nerves that are present near the aneurysm during a far-lateral approach. PICA aneurysms may be located either outside the vago-accesory triangle or within the vago-accessory triangle (suprahypoglossal triangle, infrahypoglossal triangle, or beneath the hypoglossal nerve). The study by Rodriguez and Lawton (15) demonstrated that aneurysms located outside the vago-accesory triangles were associated with no clinical deterioration and generally good overall outcomes.

Another retrospective study done by Kleinpeter et al. (16) from Austria, which included 14 cases of PICA aneurysms treated surgically between 1976 and 2003, reported that 78% of patients had favourable outcomes. In comparison, our shorter duration study with the same number of cases demonstrated that 73% of patients had a good outcome.

All three PICA aneurysm cases presented with different locations and morphologies. Therefore, the surgical approaches varied, with the most challenging strategies required when the aneurysm located at the proximal PICA. This is because the surgical field are limited, making the procedure more delicate. The approaches or choices of surgical techniques are more complex for proximal PICA aneurysms compared to distal PICA aneurysms, where simple clipping of aneurysm is easier and can be performed by junior or young neurosurgeon. In contrast, well-experienced senior neurosurgeons are required for proximal PICA aneurysm cases. Careful preoperative planning is essential, as graft harvesting (OA/STA/RAG) may be necessary if bypass is required.

### Limitations

Due to its rarity, only a limited number of PICA aneurysm cases were captured for this retrospective analysis at our centre, which may introduce bias into this study. Larger studies, possibly involving multiple centres, should be conducted to determine the most appropriate approaches that are suitable for the current available treatments and surgical expertise in Malaysia. Therefore, as few neurosurgeons are well-experienced in complex vascular surgery, the choice of surgical treatment sometimes depends on the surgeon’s capability at the time of surgery.

## Conclusion

PICA aneurysms are uncommon, and their treatments are challenging. Multiple treatment options exist, including endovascular and microsurgical approaches. Careful case selection and consideration of aneurysm characteristics are important in guiding treatment decisions. Favourable outcomes could be achieved when PICA aneurysms are treated with appropriate surgical strategies and by experienced surgeons. However, due to its rarity, many neurosurgeons have limited exposure to such cases and may lack the experience or technical skills required for microsurgical treatment of PICA aneurysms. Training programmes for neurosurgeons to equip them with the necessary skills and knowledge should be prioritised, especially in developing countries.

## Figures and Tables

**Table 1:** Summary of individual PICA aneurysm cases (*n* = 14)

Case	Age/sex	Side, segment (location), size (mm)	Morphology, complexity	Intervention	Specific procedure	surgery mRS before	mRS at discharge	
1	70/F	Left, proximal (P2) 3.2 × 2.1 × 2.0	Saccular, simple	Surgery	Far-lateral craniotomy and clipping	1	1	
2	40/F	Right, proximal (P1) 8.8 × 5.0 × 3.2	Fusiform, complex		Far-lateral craniotomy, PICA–PICA bypass and right VA ligation	5	4	
3	58/M	Right, proximal (P1) 4.4 × 2.1 × 2.0	Fusiform, complex		Right lateral suboccipital and clipping	5	6	
4	58/F	Right, distal (P5) 1.8 × 2.3 × 2.1	Saccular, simple		Suboccipital craniotomy, C1 laminectomy and clipping	1	2	
5	52/F	Left, proximal (P2) 3.0 × 2.5 × 1.4	Saccular, complex		Left far-lateral craniotomy and clipping	5	5	
	
6	21/M	Left, distal (P5) 2.0 × 3.1 × 1.5	Saccular, simple	Endovascular	Embolisation	1	0	
7	52/F	Left, distal (P5) 2.3 × 2.0 × 2.0	Saccular, complex		Embolisation	1	0	
8	39/M	Right, proximal (P1) 3.3 × 2.0 × 2.8	Dissecting, complex		Stenting	4	4	
	
9	72/F	Right, proximal (P1) 3.2 × 3.0 × 2.1	Saccular, simple	Surgery	Far-lateral craniotomy and clipping	5	1	
10	52/M	Right, mid (P3) 3.0 × 3.5 × 5.0	Fusiform, complex		Suboccipital craniotomy and clipping	1	0	
11	55/M	Left, distal (P4) 3.0 × 3.0 × 3.5	Saccular, simple		Suboccipital craniotomy and clipping	5	3	
12	48/M	Right, proximal (P2) 3.1 × 3.0 × 3.1	Saccular, simple		Far-lateral craniotomy and clipping	4	2	
13	53/M	Left, mid (P3) 3.0 × 5.5 × 10.5	Fusiform, complex		Far-lateral craniotomy and clipping	3	2	
14	39/F	Left, distal (P4) 3.3 × 2.5 × 2.2	Saccular, simple		Far-lateral craniotomy and clipping	2	2	

F = female; M = male; mRS = modified Rankin Scale

**Table 2:** Comparison of clinical outcomes according to aneurysm intervention, complexity, and location

Category	*P*-value (Fisher’s exact test)	*P*-value (Chi-square test)	Interpretation
Intervention vs. mRS outcome	1.00		No significant difference
Complexity vs. mRS outcome	0.07		No significant difference
Location vs. mRS outcome		0.06	No significant difference

**Table 3:** Changes in functional outcomes (mRS) before and at discharge (*n* = 14)

mRS before surgery	mRS at discharge		Total

	Good outcome (mRS 0 to 3)	Poor outcome (mRS 4 to 6)	
Good outcome (mRS 0 to 3)	7	0	7
Poor outcome (mRS 4 to 6)	3	4	7
Total	10	4	14
